# Inferring an Observer’s Prediction Strategy in Sequence Learning Experiments

**DOI:** 10.3390/e22080896

**Published:** 2020-08-15

**Authors:** Abhinuv Uppal, Vanessa Ferdinand, Sarah Marzen

**Affiliations:** 1 W.M. Keck Science Department, Pitzer, Scripps, and Claremont McKenna Colleges, Claremont, CA 91711, USA; auppal22@students.claremontmckenna.edu; 2 Melbourne School of Psychological Sciences, University of Melbourne, Parkville, Victoria 3050, Australia; vanferdi@gmail.com

**Keywords:** stochastic processes, prediction, Bayesian models, sequence learning

## Abstract

Cognitive systems exhibit astounding prediction capabilities that allow them to reap rewards from regularities in their environment. How do organisms predict environmental input and how well do they do it? As a prerequisite to answering that question, we first address the limits on prediction strategy inference, given a series of inputs and predictions from an observer. We study the special case of Bayesian observers, allowing for a probability that the observer randomly ignores data when building her model. We demonstrate that an observer’s prediction model can be correctly inferred for binary stimuli generated from a finite-order Markov model. However, we can not necessarily infer the model’s parameter values unless we have access to several “clones” of the observer. As stimuli become increasingly complicated, correct inference requires exponentially more data points, computational power, and computational time. These factors place a practical limit on how well we are able to infer an observer’s prediction strategy in an experimental or observational setting.

## 1. Introduction

Over the last 30 years, brains have been increasingly viewed as prediction machines. Organisms are bombarded by information, which they heavily compress and use to predict both their environment and the consequences of their actions in their environment. Cognitive systems leverage prediction to interface with the external world [1] and to internally process information within the brain [2,3]. Our current prediction-centric view of human cognition has origins in Helmholtz’s (1860) framework of perception as inference [4], later gathered momentum from applications of information theory to cognition [5], and is currently encapsulated by work on Helmholtz machines [6], the free-energy principle [7], embodied cognition [8], and Bayesian inference [9].

Bayesian modelling has proven itself to be a fruitful way to model cognition across a variety of problem domains (see Tenenbaum et al. [10,11,12] for reviews and Griffiths et al. [13] for an in-depth tutorial). Bayesian models are a useful theoretical tool because they define what the optimal ideal observer would infer in a given problem domain and allow experimentalists to compare human performance to that ideal. Humans have been shown to perform close to optimal on a variety of cognitive tasks, ranging from motor control [14], visual perception [15], motion illusions [16], pattern segmentation [17], categorization [18], word learning [19], causal inference [20], mental simulation [21], to symbolic reasoning [22]. However, often, human observers differ in interesting ways from the optimal performance of ideal observers. Suboptimal performance has been documented in motion perception [23], 3-D object recognition [24], object localization and identification [25], cue integration [26], signal detection [27], confidence reports [28], the relationship between confidence and accuracy [29], visual illusions [30], and information seeking [31]. Human performance is generally understood to be closest to optimal at the lower cognitive level of perception, but see Rahnev and Denison [32] for a review of counter-examples.

The predictive brain is nearly canon, but our current wealth of experimental and theoretical effort still begs several basic questions. Many concerns have been raised about the rather ad hoc way that ideal observers are defined, with one of the more common criticisms being that any human behavior can be shoe-horned into optimality given the right choice of priors [33]. In this paper, we introduce a more principled method for inferring an observer’s prediction strategy (i.e., prediction algorithm) using a semi-recently developed method in Streiloff et al. [34] and extend their framework so that it can be applied to experimental data from a finite number of human (or animal) observers. We focus on the general problem of inferring stochastic processes, which can be represented as a sequence learning task for the purpose of experimentation with real-world observers. We find, perhaps surprisingly, that it is difficult in a standard sequence learning experiment to uncover a single observer’s prediction strategy.

## 2. Background

### 2.1. A Hypothetical Experiment

To begin, let us consider a hypothetical sequence learning experiment, similar to Visser et al. [35]. In this experiment, a sequence of symbols is displayed one by one to a participant (i.e., observer). Each symbol presentation constitutes one trial, where $s_{t}$ denotes symbol *s* on trial *t*. These symbols come from a finite alphabet A and are generated from a order-*R* Markov model that is unknown to the observer. In this paper, we consider the special case where A is binary.

**Figure 1:**
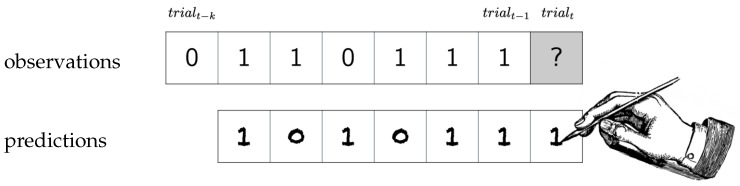
The quintessential experiment to reveal an observer’s prediction strategy: observations are shown to the observer, who then tries to predict the next observation. This happens repeatedly for as many trials as the observer can stand.

The goal of the observer is to accurately predict the upcoming symbol in the next trial (Figure 1). In order to achieve this, the observer must infer all or part of the underlying structure of the Markov model, using only the symbols observed thus far. We denote the prediction of $s_{t}$ using all prior input symbols $s_{1},\cdots ,s_{t-2},s_{t-1}$ as ${\hat{s}}_{t}$. Importantly, the observer’s prediction does not affect the next observation. This is clearly a difficult prediction task for humans, especially given our natural memory limitations. Nonetheless, a variety of prediction strategies could be used to achieve better than random performance on this task.

The goal of the experimenter, therefore, is to infer what prediction strategies the observer is using to solve this task, and to collect enough data to make this inference possible. This paper is concerned with this experimenter’s dilemma: how and how well we can correctly recover participants’ prediction strategies, given various amounts of finite data? To address this question, we specify some hypothetical observers (where their true prediction strategy is known) and assess how well we can correctly recover these strategies given only two types of data: the sequence of symbols they observed and the sequence of predictions they made.

### 2.2. Some Hypothetical Observers

We entertain three types of hypothetical observers, which from here forward we will refer to by their prediction strategy: n-gram average, n-gram argmax, and generalized linear model. The first two strategies are observers that use Bayes’ theorem to calculate a posterior of the model topology given data, and then maximize the posterior to infer the order-*R* Markov model that best fits the sequence they observed. The third strategy is a generalized linear model that does not infer the underlying structure of the Markov model, but rather relies on superficial regularities in the sequence of symbols to make its predictions. All three observers are tasked with estimating emission probabilities (i.e., the probabilities that each symbol in alphabet A will be emitted on trial *t*) and then guessing a symbol with a frequency commensurate with its emission probability. In particular, if the observer estimates that symbol *s* will appear next with probability q(s), then the observer guesses *s* with probability q(s). This behavior is known as probability matching and was chosen to better match what humans and animals are known to do in a wide range of psychological and economic decision making experiments [36]. In the remaining part of this section, each strategy is described in more detail. To simplify notation, we also denote $s_{t-k+1}...,s_{t-1},s_{t}$ as ${\overset{\leftarrow }{s}}_{t}^{k}$.

In both n-gram strategies, an observer fits an order-*R* Markov model $M_{R}$ (a model in which only the last *R* symbols are useful for predicting the next symbol, see Section 2.3) to the input string ${\overset{\leftarrow }{s}}_{t}^{t}$ and does one of two things with the associated conditional probability $P(M_{R}|{\overset{\leftarrow }{s}}_{t}^{t})$: argmax or averaging. In the n-gram argmax strategy, the observer finds the model topology (or Markov order) and corresponding model parameters θ that best matches the input string, defining the best-fit Markov order $R^{*}$ as

(1) $$R^{*}:=\arg\max_{R}P\left(M_{R}|{\overset{\leftarrow }{s}}_{t}^{t}\right).$$

Note that $R^{*}$ depends on the entire history of the experiment, ${\overset{\leftarrow }{s}}_{t}^{t}$. This is essentially a maximum a posteriori estimation to choose the model class $M_{R}$, combined with a maximum likelihood estimation to choose parameters θ for that model class. Then, as previously stated, the n-gram argmax observer finds the most likely emission probabilities θ for that model topology or Markov order. The observer uses those emission probabilities to predict the input as described earlier. In the n-gram average strategy, the observer calculates the average emission probability across all model topologies and all sets of model parameters, and uses this average emission probability to probability match.

Each n-gram strategy is defined by three parameters. First, α (the concentration parameter) defines the observer’s prior over emission probabilities. Second, γ defines the observer’s penalty on more complicated models with more states. Both of these will be described more precisely in the following subsection. The third parameter, β, is used to model the observer’s memory limitations. In behavioral experiments, it often seems as if organisms are randomly dropping observations and not using said observations to update their model of the world [37]. We assume that this happens with probability β.

In generalized linear model strategies, emission probabilities are nonlinear functions (e.g., sigmoids for binary alphabet processes) of linear weightings of a finite number *k* of prior symbols. Clearly, there will be some stimuli for which the generalized linear model strategies will be woefully inadequate. For instance, consider a simple binary-alphabet order-2 Markov stimulus for whom the probability of emitting $s_{t+1}$ given $s_{t},\;s_{t-1}$ is some nonlinear function of $as_{t}+bs_{t-1}+cs_{t}s_{t-1}$. If *c* is nonzero, then a generalized linear model cannot completely predict the stimulus as well as possible, even with infinite data.

In order to make predictions, our simulated Bayesian observers will need to use a principled approach to modeling time series, which are realizations of what are known as stochastic processes. The principled approach employed in this paper is based on knowledge of order-*R* Markov models, which we describe in the next section.

### 2.3. Memory, Complexity, and Order-*R* Markov Models

The complexity of a time series can be described by how much memory one needs in order to predict the future as well as possible. One way of quantifying this memory is to ask how many symbols in the past are required to predict the future as well as possible. The number, *R*, of such symbols is the Markov order of the time series. We explain Markov order by starting with the simplest case: Markov order 0.

**Figure 2:**
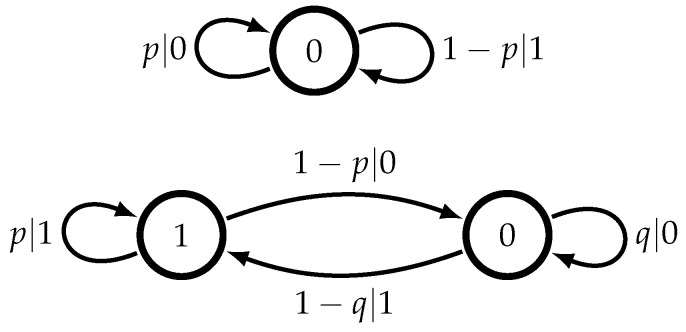
Two example order-*R* Markov models. Order 0 (**top**) and 1 (**bottom**) with the finite alphabet A={0,1}.

The reader might be familiar with order-0 Markov models which, in our binary-alphabet setup, are essentially biased coin flips. A diagram of such a model is shown in Figure 2 (top), where an outcome of heads (0) occurs with probability *p* and tails (1) occurs with probability 1−p. An order-0 Markov model is a memoryless process consisting of one state (the node) with all transitions (the arrows) leading back into itself. This is too simple a case to gain insight into order-*R* Markov models, so we proceed to order-1 Markov models, usually just called “Markov models”.

Markov models generate stochastic processes in which only the present symbol is needed to understand the future. Prior symbols contain no information beyond that contained in the present symbol. Formally, if we denote the symbol at time *t* as $s_{t}$, and the history at time *t* as $h_{t}=\left\{s_{1},s_{2},...,s_{t}\right\}$, which as described earlier, we write as ${\overset{\leftarrow }{s}}_{t}^{t}$:

(2) $$P\left(s_{t+1}|h_{t}\right)=P\left(s_{t+1}|s_{t}\right).$$

Note that, of course, most time series do not satisfy the Markov property. Usually, things far in the past have some effect on the future.

Although we have defined a Markov model by the statistics of the process that it generates, one can also understand Markov models via diagrams similar to those shown in Figure 2 (bottom). Note that all arrows that end on state 1 imply an emission of the symbol 1, and similarly for 0. Hence, the states are “visible”: knowing the present state defines the next symbol. These states are exactly what one needs to know in order to predict the future as well as possible.

A more complex time series will have finite Markov order *R* greater than 1, meaning that

(3) $$P\left(s_{t+1}|h_{t}\right)=P\left(s_{t+1}|{\overset{\leftarrow }{s}}_{t}^{R}\right).$$

Note that now the last *R* symbols affect our predictions, and so predicting as well as possible requires storing those last *R* symbols. In psychology, this is equivalent to an *n*-gram strategy where n=R. Hence, in principle, an observer that uses an *n*-gram strategy for prediction should be able to understand an order-*R* Markov stimulus.

Again, intuitively, the order of a Markov model *R* quantifies the memory of the process. This model has states defined by the last *R* symbols outputted by the model. Having a higher-order Markov model is tantamount to having a process with a more detailed structure, which corresponds to a larger number of states. In particular, if we denote our alphabet size as |A|, then the number of transitions in an order-*R* Markov model is ${\left|A\right|}^{R}\left(|A|-1\right)$. This exponential relationship between the size of the model and the order of the Markov model can make computation of nearly anything (entropy rates, the most likely model, etc.) difficult.

To infer the order-*R* Markov model that best fits a time series in a principled manner, one tries to maximize the conditional probability distribution over models given the input ${\overset{\leftarrow }{s}}_{t}^{t}$. There are two aspects to this model, when viewed graphically as a set of nodes (histories of length *R*) that you transition between based on symbols while emitting symbols probabilistically. The first aspect is the order of the model *R*, as this determines the model “topology”; we denote this variable via $M_{R}$. The second is the estimated transition probabilities of emission at each node; we denote all of these transition probabilities as a parameter θ, though each transition probability is given by $p(s_{t}|{\overset{\leftarrow }{s}}_{t-1}^{R})$. The best-fit model is thus fully specified by the posterior $P(M_{R},\theta |{\overset{\leftarrow }{s}}_{t}^{t})$, which our theoretical observers will use as described in Section 2.2. Though calculating this posterior is quite difficult, it can be done analytically [34] when you choose a Dirichlet distribution as the prior for the model, so that

$$P\left(\theta |M_{R}\right)=\frac{1}{Z}\delta \left(1-\sum_{s_{t}}p\left(s_{t}|{\overset{\leftarrow }{s}}_{t-1}^{R}\right)\right)\prod_{s_{t},{\overset{\leftarrow }{s}}_{t-1}^{R}}p{\left(s_{t}|{\overset{\leftarrow }{s}}_{t-1}^{R}\right)}^{\alpha -1},$$

where α is known as the concentration parameter. This concentration parameter might vary from observer to observer. One can also specify a prior distribution over model topologies or equivalently over Markov orders via $P\left(M_{R}\right)=e^{-\gamma (|A|-{1)|A|}^{R}}$, where γ might vary from observer to observer as well. As described in the appendix, once you choose this prior, one can calculate the posterior as

$$P\left(M_{R}|{\overset{\leftarrow }{s}}_{t}\right)=e^{-{\gamma |A|}^{R}}\prod_{{\overset{\leftarrow }{s}}^{R}\in A^{R}}\frac{\Gamma (n\left({\overset{\leftarrow }{s}}^{R}\right)+|A|\alpha )}{\prod_{s\in A}\Gamma \left(\alpha \right)}\frac{\prod_{s\in A}\Gamma \left(n\left({\overset{\leftarrow }{s}}^{R}s\right)+\alpha \right)}{\Gamma (n\left({\overset{\leftarrow }{s}}^{R}\right)+|A|\alpha )},$$

in which n(·) refers to the number of times that a particular string · has been observed in the history of the experiment (last *t* input symbols).

## 3. Results

In our hypothetical sequence learning experiment, symbols are shown to an observer or to an array of identical observers. These observers are asked to predict the next symbol. Our task is to infer the observers’ prediction strategy from the input symbols and their predictions.

In order to infer the observer model, we calculate the likelihood of seeing the given stream of predictions and maximize this likelihood with respect to the observer model. The explicit mathematical setup is explained in Appendix A.

This section proceeds in three parts. First, we develop new closed-form expressions for the likelihood of a particular observer model given the input dataset and predictions, so that a maximum likelihood approach to inferring observer strategy can be easily employed. Next, we show that when we have access to an arbitrarily large number of identical observers, we can correctly infer the observers’ prediction strategy quite well. When only one observer is present, we can infer the model class (generalized linear model vs. Bayesian model), but not the parameters of the model.

### 3.1. A Simple, Principled Strategy for Inferring an Observer’s Prediction Algorithm

The question remains as to how exactly we will attempt to infer the prediction strategy of the observer *O*, whether it be n-gram argmax or n-gram average or GLM. The basic idea is simple: we calculate the probability that a particular observer could have generated the string of observations and the string of predictions seen. We then find the observer strategy that maximizes this likelihood,

(4) $$O^{*}=\arg\max_{O}P\left({\overset{\leftarrow }{s}}_{t}^{t},{\overset{\leftarrow }{\hat{s}}}_{t}^{t}|O\right).$$

We use L to denote $P({\overset{\leftarrow }{s}}_{t}^{t},{\overset{\leftarrow }{\hat{s}}}_{t}^{t}|O)$. The maximum likelihood $O^{*}$ represents our best guess as to which observer model describes the predictor. For example, $O^{*}$ might be an n-gram argmax with α=1,β=0.5,γ=2, or $O^{*}$ might be an n-gram average observer with α=1,β=0.5,γ=2. Note that $O^{*}$ depends on not only the input signals $s_{t}$ but also the predictions ${\hat{s}}_{t}$.

Although maximum likelihood methods sometimes run into issues with nuisance parameters that are better treated by maximum a posteriori methods, we take the prior over observer models to be uniform. That, combined with the fact that our likelihoods are highly peaked at one particular value, result in a correspondence between maximum likelihood and maximum a posteriori. Future applications should be wary of using our methods if observer models have a proliferation of parameters.

Maximum likelihood estimation is certainly not a new idea. However, actually evaluating the likelihood and maximizing it can be difficult. In this subsection, we report formulae for the likelihood of the n-gram argmax and n-gram average strategies.

For the n-gram argmax observer, the log likelihood is given by

(5) $$\log\left(L\right)=\sum_{t}\log P_{ML}\left({\hat{s}}_{t+1}|M_{ML,t}\right)$$

where $P_{ML}\left({\hat{s}}_{t+1}|M_{ML,t}\right)$ is the estimated probability by an order-R* Markov modeler of next seeing ${\hat{s}}_{t+1}$. (Note the conversion of $P({\overset{\leftarrow }{s}}_{t}^{t},{\overset{\leftarrow }{\hat{s}}}_{t}^{t}|O)$ to a product of conditional probabilities, hence the sum over time *t*.) For us, $R^{*}$ can be calculated using the analytic expressions in Ref. [34], as given also in the appendix for completeness. In the appendix, we evaluate this probability as

(6) $$P_{ML}\left({\hat{s}}_{t+1}|{\overset{\leftarrow }{s}}_{t}^{R}\right)=\frac{\alpha +\beta n({\overset{\leftarrow }{s}}_{t}^{R}{\hat{s}}_{t+1})-1}{2\alpha +\beta n({\overset{\leftarrow }{s}}_{t}^{R})-2}.$$

The most likely model (ML) is the order-$R^{*}$ Markov model with

(7) $$\begin{array}{ccc} R^{*} & = & \arg\max_{R}P\left(M_{R}|{\overset{\leftarrow }{s}}_{t}^{t}\right)., \end{array}$$

as described in Section 2. Whereas for the n-gram average strategy, the log likelihood is similarly

(8) $$\log\left(L\right)=\sum_{t}\log\langle P\left({\hat{s}}_{t+1}|M_{R,t}\right)\rangle$$

where $\langle P\left({\hat{s}}_{t+1}|M_{R,t}\right)\rangle$ is the probability of observing ${\hat{s}}_{t+1}$ under each possible order-*R* Markov model, averaged over all order-*R* Markov models. In other words,

(9) $$\langle P\left({\hat{s}}_{t+1}|M_{R,t}\right)\rangle =\sum_{M}\;\left\{\int d\theta P_{t}\left(\theta ,M\right)P\left({\hat{s}}_{t+1}|{\overset{\leftarrow }{s}}_{t}^{R},\theta ,M\right)\right\}$$

which, after straightforward manipulation shown in the appendix, reduces to

(10) $$\langle P\left({\hat{s}}_{t+1}|M_{R,t}\right)\rangle =\sum_{R}\left(P_{0}\left(M_{R}\right)\frac{\alpha +\beta n({\hat{s}}_{t+1}|{\overset{\leftarrow }{s}}_{t}^{R})}{2\alpha +\beta n({\overset{\leftarrow }{s}}_{t}^{R})}\right).$$

The prior $P_{0}\left(M\right)$ is our prior distribution over models, i.e., $e^{-{\gamma |A|}^{R}\left(|A|-1\right)}$. Altogether, we have

(11) $$\log\left(L\right)=\sum_{t}\ln\left(\sum_{R}\exp{\left[-\gamma |A\right|}^{R}\left(|A|-1\right)\right]\frac{\alpha +\beta n({\hat{s}}_{t+1}|{\overset{\leftarrow }{s}}_{t}^{R})}{2\alpha +\beta n({\overset{\leftarrow }{s}}_{t}^{R})})$$

where ${\overset{\leftarrow }{s}}_{t}^{R}$ is the previous *R* symbols. Details of the derivation are in Appendix A.

The GLM log likelihood does not need similar analytic manipulation as it does not require an integral over all possible models. For binary alphabet input, the GLM likelihood can be computed with relative ease by employing a logistic regression on the last *k* symbols, estimating the probability of observing ${\hat{s}}_{k+1}$ based on this regression, and then calculating

(12) $$\log L=\sum_{t}P_{GLM}\left({\hat{s}}_{k+1}|{\overset{\leftarrow }{s}}_{k}^{k}\right),$$

where $P_{GLM}\left({\hat{s}}_{k+1}|{\overset{\leftarrow }{s}}_{k}^{k}\right)$ is the aforementioned estimated probability.

To infer the observer model, we simply find the combination of parameters that maximizes the log likelihood, then choose the maximal log likelihood between n-gram argmax, GLM, and n-gram average strategies. When there are multiple identical observers, as in the next subsection, we average the log likelihood over observers and find the parameters and strategy that maximizes this average log likelihood.

Both of the n-gram log likelihoods are parameterized by three exogenous numbers: {α,β,γ}. A natural next question is to ask whether or not this observer inference scheme actually works. The following sections address this.

### 3.2. With Infinite Identical Observers, We Can Infer Observers’ Prediction Strategies

We first examine a somewhat unrealistic situation in which we have an arbitrarily large number of identical observers all predicting (independently) the next symbol at each time step, for an arbitrarily large number of time steps. We begin with this situation because it is the worst-case scenario for an experimenter: if we can not correctly infer prediction strategies in this case, then we can not do any better given less data. Additionally, it can be reasonable to assume identical observers in cognitive domains where participants have the same prior, such as certain low-level perceptual tasks, or when trying to explain the performance of a deep learning system, which has an architecture that could be cloned an arbitrarily large number of times and run for an arbitrarily large number of time steps.

It will turn out to be the case that our ability to distinguish between different observer strategies is entirely governed by the emission probabilities of each of the strategies, as defined earlier. To that end, we provide a sketch of a proof for a useful lemma below.

**Lemma** **1.** *Consider an infinite string of symbols that is generated by an infinite-order Markov stimulus. The probabilities ${\left\{p_{obs}\left({\hat{s}}_{t+1}|{\overset{\leftarrow }{s}}_{t}^{t}\right)\right\}}_{t=1}^{\infty }$—the probabilities that a particular observer guesses ${\hat{s}}_{t+1}$ given that she has seen ${\overset{\leftarrow }{s}}_{t}^{t}$—uniquely define the observer strategy up to a multiplicative constant.*

**Proof.**  The essence of this proof is that the emission probabilities for each strategy are governed by a finite number of parameters. For n-gram observers, only three parameters are needed; for generalized linear model observers, only *k* parameters are needed, where *k* is an unspecified but finite integer. However, matching emission probabilities for an arbitrarily large number of steps requires satisfying an arbitrarily large number of equations. With finite parameters but infinite equations, there is almost always no solution.

Let us review which equations we have to match.

For the n-gram argmax, we have

$$p_{obs}\left({\hat{s}}_{t+1}|{\overset{\leftarrow }{s}}_{t}^{t}\right)=\frac{\alpha +\beta n({\overset{\leftarrow }{s}}_{t}^{R^{*}}{\hat{s}}_{t+1})-1}{|A|\left(\alpha -1\right)+\beta n\left({\overset{\leftarrow }{s}}_{t}^{R^{*}}\right)}$$

where $R^{*}$ is the Markov order that maximizes the posterior probability over such orders. Though the actual equation for the posterior probability is somewhat messy, in the large time limit, one can straightforwardly show that $R^{*}$ satisfies

$$R^{*}\approx \arg\max_{R}-\left(|A\right|-{1)|A|}^{R}\gamma +{\left|A\right|}^{R}\log\frac{\Gamma (|A|\alpha )}{\Gamma {\left(\alpha \right)}^{\left|A\right|}}+th_{\mu }\left(R\right).$$

The term $h_{\mu }\left(R\right)$ is shorthand for the conditional entropy of the R+1 symbol given the previous *R* symbols, $H[S_{R+1}|S_{1},...,S_{R}]$ [38]. This conditional entropy, by definition, can only decrease as *R* increases. As a result, as *t* increases, once *t* is large enough, $R^{*}$ always increases, since the stimulus is in fact infinite-order Markov, and so higher orders will always fit the data better. Furthermore, $R^{*}$ is also highly dependent on the prior γ; a stronger insistence on simpler models will cause $R^{*}$ to decrease. Note that while α and β obviously control the emission probabilities, γ only indirectly controls emission probabilities by controlling $R^{*}$.

For the *n*-gram average, we have

$$p_{obs}\left({\hat{s}}_{t+1}|{\overset{\leftarrow }{s}}_{t}^{t}\right)=\frac{1}{Z}\sum_{R=0}^{\infty }e^{-\gamma (|A|-{1)|A|}^{R}}\frac{\alpha +\beta n({\overset{\leftarrow }{s}}_{t}^{R}{\hat{s}}_{t+1})}{\left|A|\alpha +\beta n({\overset{\leftarrow }{s}}_{t}^{R}\right)},$$

where *Z* is a normalization factor given by $Z=\sum_{R=0}^{\infty }e^{-\gamma (|A|-{1)|A|}^{R}}$. Here, γ more obviously affects the emission probabilities.

At this point, we stop to remark on a crucial difference between the emission probabilities of the two n-gram strategies. In n-gram argmax, the emission probabilities are simply a linear function of α and β divided by a different linear function of α and β. In n-gram average, the emission probabilities are also rational functions, but with unbounded degree. As *t* advances, we alter the coefficients of the rational functions in a semi-random way. It is impossible for the two emission probabilities to match unless there is a fortuitous cancellation of higher coefficients for all times *t*, which corresponds to a measure-0 subset of all possible sequences. In other words, it is always possible to distinguish the two n-gram strategies from one another if there are enough observers and enough time steps.

Similarly, we can distinguish identical strategies with different parameters from one another up to a multiplicative constant. The key here is not a difference in the type of equation (rational with high degree versus low degree) but an overdetermined set of equations. In order for one n-gram argmax strategy to be confused with another, one must satisfy an infinite number of linear equations with two parameters. For example, suppose in a proof by contradiction that an n-gram argmax observer with α,β was confused with an n-gram argmax observer with $\alpha^{\prime },\;\beta^{\prime }$. We would have to satisfy

$$\frac{\alpha +\beta n({\overset{\leftarrow }{s}}_{t}^{R^{*}}{\hat{s}}_{t+1})-1}{|A|\left(\alpha -1\right)+\beta n\left({\overset{\leftarrow }{s}}_{t}^{R^{*}}\right)}=\frac{\alpha^{\prime }+\beta^{\prime }n\left({\overset{\leftarrow }{s}}_{t}^{R^{*}}{\hat{s}}_{t+1}\right)-1}{\left|A\right|\left(\alpha^{\prime }-1\right)+\beta^{\prime }n\left({\overset{\leftarrow }{s}}_{t}^{R^{*}}\right)}$$

for all time *t*. Some manipulation shows that this is only possible if $\beta /\left(\alpha -1\right)=\beta^{\prime }/\left(\alpha^{\prime }-1\right)$, so β and α can be distinguished up to a multiplicative constant. The parameter γ, once this multiplicative constant is specified, uniquely follows, as $R^{*}$ (which controls $p_{obs}$) is determined by α,γ, as long as $\left(|A|-1\right)\gamma -\log\left(\frac{\Gamma (|A|\alpha )}{\Gamma {\left(\alpha \right)}^{\left|A\right|}}\right)$ is at the right value. Similar logic holds for n-gram average, so that the probabilities are only matched if β/α is held constant, i.e., α and β are uniquely determined up to a multiplicative constant. For n-gram average, unlike n-gram argmax, γ is not controlled by this multiplicative constant.

Finally, we briefly touch upon the generalized linear model strategies. The key here is that the generalized linear models are constricted to have a finite number of parameters, making them unable to correctly model emission probabilities as *t* grows, whereas the n-gram strategies continue to improve by shifting their order higher and higher. Since the stimulus is infinite-order Markov, there is no chance that the generalized linear model emission probabilities can match those of the n-gram observers for all times *t*. □

The lemma above implicitly has three very limiting assumptions that are worth remarking on.

First, in both n-gram argmax and n-gram average, the parameters α,β and (for n-gram argmax) γ are determined up to a multiplicative constant. In n-gram argmax, we can scale both β and α−1 by a constant *L* and can then add $\frac{1}{|A|-1}\log\left(\frac{\Gamma (|A|L\alpha )}{\Gamma {\left(L\alpha \right)}^{\left|A\right|}}\right)-\frac{1}{|A|-1}\log\left(\frac{\Gamma (|A|\alpha )}{\Gamma {\left(\alpha \right)}^{\left|A\right|}}\right)$ to γ without any change in likelihood. Hence, in the future, for n-gram argmax inference results, we report the ratio

(13) $$\phi_{ngram-argmax}:=\frac{\beta }{\alpha -1}$$

as a way of testing the quality of the parameter inference. Similarly, in n-gram average, both α and β can be rescaled by *L* with no change in likelihood, and so we report

(14) $$\phi_{ngram-average}:=\frac{\beta }{\alpha }$$

to test the quality of the parameter inference. This means that even with infinite data, infinite identical observers, and an infinitely complex stimulus, the exact parameters of the observer’s prediction algorithm cannot be inferred precisely. As we shall see in a later subsection, this does not prevent us from correctly inferring the general strategy, or inferring the parameter values up to a multiplicative factor.

Second, the lemma above demands that the emission probabilities for all times *t* exactly match, not just approximately match. Without this stipulation, the two n-gram strategies would usually be deemed equivalent, as the posterior probability distribution over order is usually very highly peaked. With a finite number of identical observers, it does not make sense to demand that emission probabilities match exactly, since your ability to detect such differences is marred by “noise”.

Third, if the stimulus is not generated by something that is infinite-order Markov, then there is a risk (however small) of the generalized linear model strategy matching either n-gram strategy. By specializing to infinite-order Markov stimuli, we can dismiss the possibility that the weakly expressive generalized linear model strategy can capture as much predictive information as the n-gram strategies. Indeed, this realization helps guide experimental design.

However, with this lemma in hand, we are poised to prove our main theorem– that of consistency of our maximum likelihood estimator.

**Theorem** **1.** *An arbitrarily long string of symbols generated by an infinite-order Markov model is shown to infinite identical observers, who at each point try to predict the next symbol. Our maximum average log likelihood prediction strategy is the true observer prediction strategy (up to the aforementioned multiplicative constant on parameters of n-gram strategies).*

**Proof.**  One can show that the average log likelihood at time step *t* takes the form of a cross-entropy:

(15) $$\langle \log L_{t}\rangle =\sum_{{\hat{s}}_{t+1}}p_{obs}\left({\hat{s}}_{t+1}|{\overset{\leftarrow }{s}}_{t}^{t}\right)\log p_{model}\left({\hat{s}}_{t+1}|{\overset{\leftarrow }{s}}_{t}^{t}\right),$$

where $p_{obs}$ is the observer’s predicted probability of seeing a particular symbol next given all previous symbols, and $p_{model}$ is the probability under a particular observer model of seeing a particular symbol next given all previous symbols. The $p_{obs}$ is obtainable from the frequency with which observers guess a particular symbol. This average log likelihood is maximized when $p_{obs}$ exactly matches $p_{model}$. In other words, unsurprisingly, average log likelihood is uniquely maximized when the prediction probabilities for the real observer matches the prediction probabilities for the inferred observer:

(16) $$p_{obs}\left({\hat{s}}_{t+1}|{\overset{\leftarrow }{s}}_{t}^{t}\right)=p_{model}\left({\hat{s}}_{t+1}|{\overset{\leftarrow }{s}}_{t}^{t}\right),\forall t\in \left[1,\infty \right)$$

We have shown in the previous lemma that these equations for a sufficiently complex stimulus only hold when the inferred observer model matches the true observer model. Hence, a maximum likelihood approach can (with enough data) reveal observers’ prediction strategy up to a multiplicative constant on parameters for n-gram strategies, as described in Lemma 1. □

The theorem above applies in the infinite data limit. Perhaps surprisingly, we can accurately infer observer prediction strategies even with a finite amount of data. In simulations of the n-gram average prediction strategy, we are able to consistently infer the correct parameters ($\phi_{ngram-average},\;\gamma$) when averaging over observer predictions, even for relatively small *t* and regardless of the model complexity. However, in simulations of n-gram argmax, however, having a limited amount of data induces some error in inferring α,β,γ up to the constant mentioned in Lemma 1. Additionally, even given this constant, error is much more prevalent in γ, since a range of γ values can produce the exact same model likelihood when given a finite string of data. A more detailed description of these results is given in the text and figures below.

We consider simulated experiments in which an order-*R* Markov stimulus is sent to infinite identical observers, who then make *N* successive predictions; from the stimulus and predictions, we use maximum likelihood estimation to infer not only the model class of the observer’s prediction strategy (generalized linear model, n-gram argmax, n-gram average) but also the parameters of each strategy. We focus primarily on the ratios $\phi_{ngram-argmax},\;\phi_{ngram-average}$ as indicators of success in inference because we can only determine our original three parameters (α,β,γ) up to a multiplicative constant.

**Figure 3:**
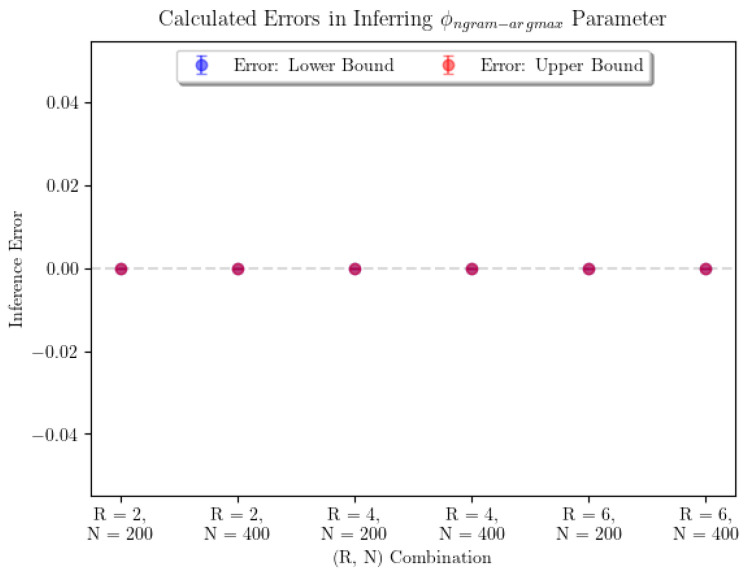

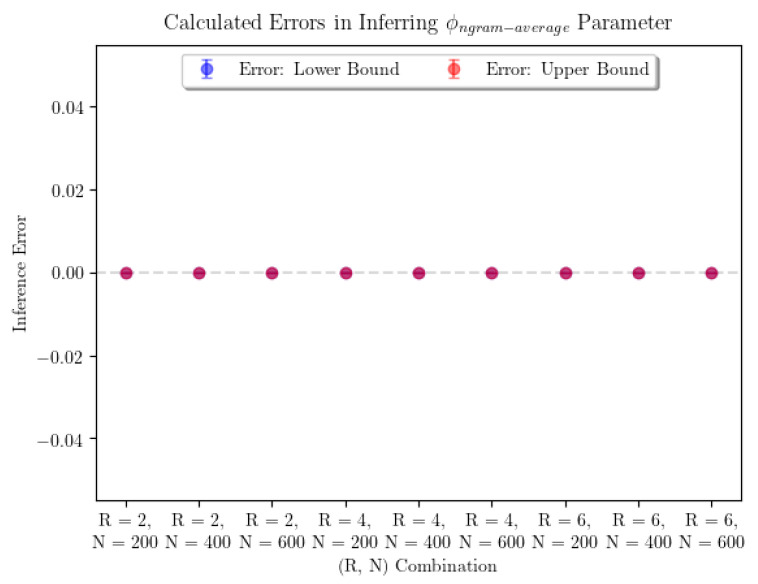
The average error in $\phi_{ngram-argmax}$ parameter inference for n-gram argmax prediction strategy and $\phi_{ngram-average}$ for the n-gram average prediction strategy shows that the inference seems to perform perfectly. For n-gram average, we see that every combination of parameters was able to produce perfect estimates for $\phi_{ngram-average}$. For n-gram argmax, the sample sizes were 7 for each pair corresponding to R=2,6 and 10 for R=4. For n-gram average, the sample sizes were 9 for each (R,N) pair.

Note that this setup breaks from the assumptions of the consistency theorem (Theorem 1) in two important ways. First, the stimulus is not infinite-order Markov. Second, only a finite number of predictions are made. The results of our inference are shown in Figure 3. In order to account for the potential existence of many global maxima, the plot shows the average error on the smallest and largest inferred values of the parameter of interest, denoted “Lower Bound” and “Upper Bound” errors, respectively. Error bars represent 95% confidence intervals for the true average parameter inference error in that particular (R,N) combination, where *R* represents the Markov order of the input and *N* the number of predictions made by observers. Realistically, if we needed to pick the observer’s “true” parameter value, we would be justified in picking any parameter value in the range between the points represented by the lowest point on the “Lower Bound” error bar and the highest point on the “Upper Bound” error bar. In this case, we were able to infer each of the ϕ parameters with no error.

**Figure 4:**
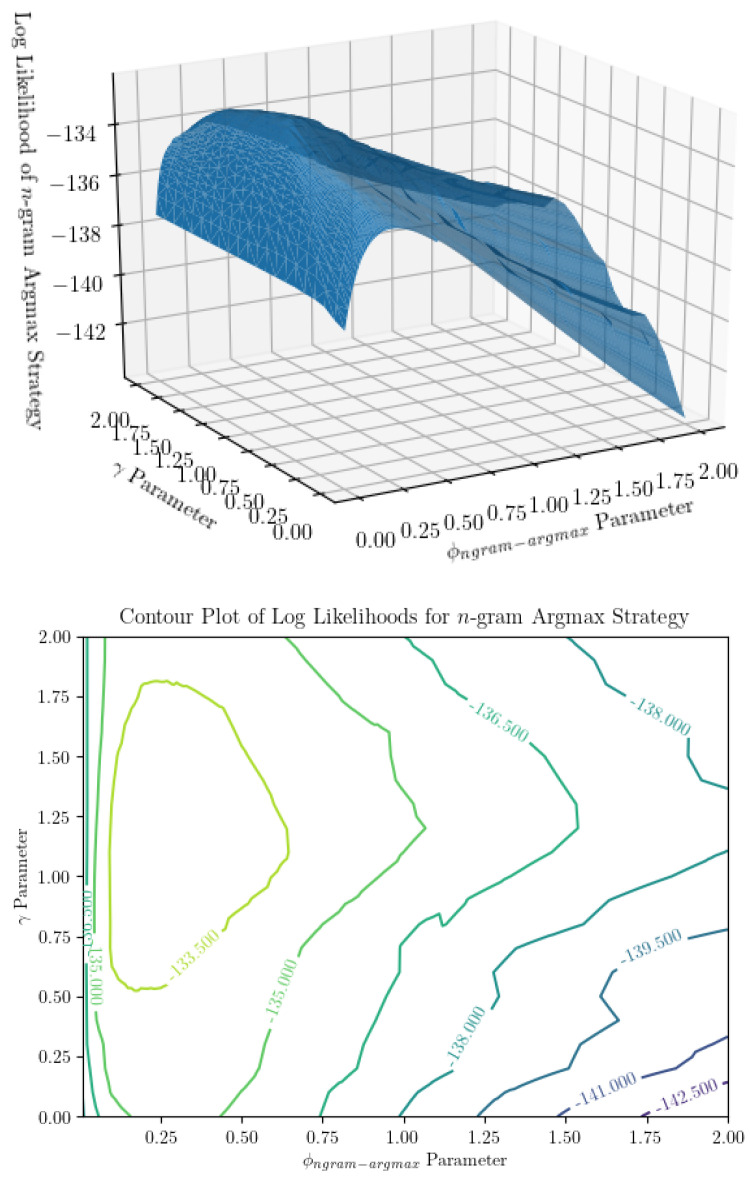
Surface and contour plot of log likelihood of the n-gram argmax prediction strategy show a peak at some combination of parameters. On the *x* and *y* axes are parameters $\phi_{ngram-argmax}$ (the ratio of β, the probability of dropping an observation, to α−1, the concentration parameter subtracted by 1) and γ (the regularization term in the prior over models), and on the *z*-axis is the log likelihood of the observer model for a string of inputs, averaged over infinite identical observers. It is difficult to see in the pictures, but there are ridges in the average log likelihood as a function of $\phi_{ngram-argmax}$, which we still cannot explain.

**Figure 5:**
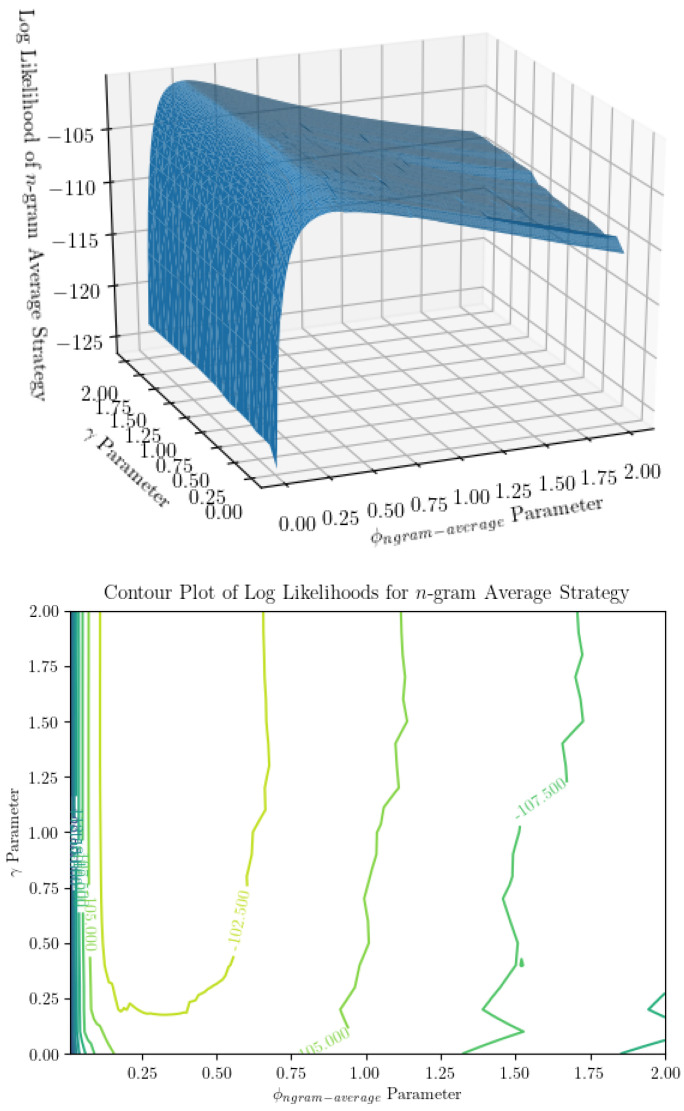
Surface and contour plot of log likelihood of the n-gram average prediction strategy show a much smoother surface than n-gram argmax. On the *x* and *y* axes are parameters $\phi_{ngram-average}$ (the ratio of β, the probability of dropping an observation, to α, the concentration parameter subtracted by 1) and γ (the regularization term in the prior over models), and on the *z*-axis is the log likelihood of the observer model for a string of inputs, averaged over infinite identical observers. This appears to be a much nicer surface to optimize over, though it is not without its ridges.

In a practical sense, computational time complexity is a large limiting factor in parameter inference for the *n*-gram argmax strategy. Examining Figure 4 and Figure 5, we can see that the surfaces representing the strategy likelihoods when parameters are varied are not convex in general. They are typically lined with many small ridges that cause numerical optimization algorithms to find optimal solutions to have trouble finding the global extrema. Thus, in order to identify the optimal parameters, we are forced to turn to a grid search. Not helping the issue is the fact that, when not in the large-sample limit, the global maximum may not be unique—searching over all parameter combinations may yield many “optimal” combinations, in the sense that they produce the maximum likelihood estimate of the prediction strategy. It’s worth noting that only being able to determine α and β up to a multiplicative constant is not an issue here, as using the ratio $\phi_{ngram-argmax}$ accounts for this. From Figure 3, we can see that using a grid search, $\phi_{ngram-argmax}$ is determined with minimal error. Finally, using a grid search limits our desired precision through the runtime. The optimization itself is incredibly computationally expensive—a time complexity of at least $O(\nu N^{2}2^{R})$—where ν is the size of the parameter space being searched over. This is a large reason we are only able to show results for a small number of iterations of parameter inference for each (R,N) combination. Additionally, increasing the precision by one decimal place when searching over two parameters will increase the size of the parameter space 100-fold. Performing parameter inference of relatively more involved stimuli (R≥6) 3 times with more than two decimal points of precision on each parameter could take upwards of several weeks to compute depending on the machine being used.

### 3.3. With Reasonable Amounts of Finite Data, We Can Only Infer the Model Class

In a behavioral experiment with human participants, 500 symbols would be a large but reasonable stimuli set; 5000 symbols would be prohibitive. With that in mind, we analyze whether or not it is possible to infer the observer’s prediction strategy with a reasonable number of observations.

In an experimental setting, we will not be able to average over observer predictions—rather, we will have to use a single string of predictions and calculate the likelihood that the observer used that prediction strategy, using the set of model parameters that produce a maximum likelihood estimate. If the special case that the prior over the observer model being either n-gram average, n-gram argmax, and GLM is uniform, if the prior over hyperparameters within each strategy is uniform, and if the likelihood over parameters for each strategy is highly peaked, then our maximum likelihood approach is essentially equivalent to maximum a posteriori. In practice, n-gram argmax and n-gram average, though different strategies, produce nearly identical likelihoods, so the question reduces to whether we can infer if the observer is using a Bayesian strategy or a generalized linear model.

When considering only a single observer’s predictions, we are able to perfectly classify whether they are using a Bayesian or GLM prediction strategy (Table 1). However, we are not generally able to infer the exact parameters governing an observer’s prediction strategy.

## 4. Discussion

The predictive brain is the dominant framework in cognitive science today, viewing humans or other animals as prediction machines. However, it seems to the authors that better tools are now available for inferring the prediction strategies of organisms and should be used to assess if and how various organisms are prediction machines. For instance, Visser et al. [35] conducted a seminal experiment much like the hypothetical sequence learning experiment in our setup. The difference was mostly that of analysis: (1) they fitted humans’ predictions to the correct hidden Markov model topology, thereby potentially biasing the results towards the conclusion that participants exhibited the correct prediction strategy, and (2) they exclusively analysed participant’s predictions and omitted the input sequence they observed, which is a rich source of trial-by-trial information for assessing an observer’s prediction strategy. Using the recent results of Strelioff and Crutchfield [34], we were able to develop an entirely new methodology for inferring the observer’s prediction strategy that takes into account the input to observers and, in theory, does not bias one towards concluding that the observer has any particular prediction strategy.

Applying our new method for inferring prediction strategy, we find that we can correctly infer said strategy when given an infinite number of identical observers. In addition, we prove a consistency theorem stating that, in the large data limit, we will always be able to identify the observers’ prediction strategy. Surprisingly, our simulations show that we can narrow down estimates of parameters perfectly for both *n*-gram strategies even with less than 500 observations, which is good news as sequence learning experiments typically obtain 100–500 observations per testing block. Unfortunately, many identical observers are necessary for accurate parameter inference. When we apply our analyses to a more realistic experimental situation, where we have a finite number of participants who exhibit individual differences in prediction strategy, things break down. In the case of one distinct observer, we are able to infer the prediction strategy well, but we are not able to infer the parameters governing either Bayesian inference strategy.

These results—that with only one observer we can infer the model class (generalized linear model vs. Bayesian model), but not necessarily the model’s parameters—are hopeful for further work and experimentation. Some ways forward may be to focus on domains in cognitive science where participants exhibit relatively little variation between individuals, such as low-level perceptual tasks. In these cases, our new methodology should be able to infer participants’ prediction strategy down to the governing parameters. However, in most other cases where participants do exhibit detectable individual differences, we are not guaranteed to produce any inferences deeper than the class of strategy itself.

## Figures and Tables

**Table 1:** The confusion matrix for strategy inference shows that we are able to perfectly infer the observer’s prediction model class, even if we are not able to perfectly infer the exact parameters.

		Actual Strategy		
		Bayesian	GLM	Total
Inferred Strategy	Bayesian	100	0	100
	GLM	0	100	100
	Total	100	100	200

## Abbreviations

The following abbreviations are used in this manuscript:

TLA	Three letter acronym
LD	linear dichroism

## Appendix A. Derivation for Expressions of Likelihood

As described in the main text, all that is required to calculate the likelihood is either the emission probability of ${\hat{s}}_{t+1}$ given the most likely model based on the data *D* (${\overset{\leftarrow }{s}}_{t}^{t}$); or the average emission probability of ${\hat{s}}_{t+1}$ where the average is taken over all models, weighted by the posterior probability of the model given the data *D* (${\overset{\leftarrow }{s}}_{t}^{t}$). To that end, we review the manipulations required to get the needed posterior probability over models given data, as we can easily from this posterior probability find the necessary (potentially averaged) emission probability.

Given a time series of input signals $D_{input,t}$, the observer’s first step to making a prediction is to infer what model structure produced this time series. (Note that even though this appendix only uses input signals to update the posterior, the predictions made by the observer affect the likelihood described in the main text because we are aiming to compute the likelihood of observing the next prediction given previous input symbols.) A Bayesian observer attempting to identify a time series of unknown Markov order will calculate the probability of the data being generated from a model of order *R*, denoted $M_{R}$, using Bayes’ theorem [34]:

(A1) $$P\left(M_{R}|D_{input,t}\right)=\frac{P_{0}\left(M_{R}\right)}{P(D_{input,t})}\;P\left(D_{input,t}|M_{R}\right)$$

Note that while generally $P(D_{input,t})\neq 1$, this is simply a normalization factor that can be ignored when comparing model posteriors as it is the same for all models. We use M to denote the set of all possible models later on.

The $P_{0}\left(M_{R}\right)$ term is the prior over models, which we assume are exponentially decreasing in the model size, regularized by a factor γ∈[0,∞):

(A2) $$P_{0}\left(M_{R}\right)=\exp\left[-\gamma |M_{R}|\right]$$

where

(A3) $$|M_{R}{\left|=|A\right|}^{R}\left|A-1\right|.$$

if we take the size of the model to be the number of parameters needed to infer. As discussed in Section 2, a model parameter is a transition probability from one state to the next $P(s_{t}|{\overset{\leftarrow }{s}}_{t-1}^{R})$. We use a single parameter θ to denote a vector of transition probabilities.

As for $P(D|M_{R})$, if we assume that the the observer uses a product of Dirichlet distributions for her conjugate prior belief given the order of the Markov model, then we can obtain a closed form expression. Note that

(A4) $$P\left(D_{input,t}|M_{R}\right)=\int d\theta \;P\left(D_{input,t}|\theta ,M_{R}\right)P\left(\theta |M_{R}\right).$$

The term $P(\theta |M_{R})$ is defined below in Equation (A9). After integrating over model parameters θ we can express the prior predictive probability of the data given the model as:

(A5) $$P\left(D_{input,t}|M_{R}\right)=\prod_{{\overset{\leftarrow }{s}}^{R}\in A^{R}}\;\left\{\frac{\Gamma (|A|\alpha )}{\prod_{s\in A}\Gamma \left(\alpha \right)}\;\frac{\prod_{s\in A}\Gamma (\beta n\left({\overset{\leftarrow }{s}}^{R}s\right)+\alpha )}{\Gamma (\beta n\left({\overset{\leftarrow }{s}}^{R}\right)+|A|\alpha )}\right\}$$

where α is the concentration parameter, $\Gamma \left(z\right)=\int_{0}^{\infty }x^{z-1}e^{-x}dx=\left(z-1\right)!\;\forall z\in Z^{+}$ is the gamma function, and $A^{R}$ is the set of all words of length *R* consisting of letters drawn from the alphabet A. (This is equivalent to the states of an order-R markov model.) In our case A={0,1}⟺|A|=2, so Equation (A5) simplifies to:

(A6) $$P\left(D_{input,t}|M_{R}\right)=\prod_{{\overset{\leftarrow }{s}}^{R}\in A^{R}}\;\left\{\frac{\Gamma (2\alpha )}{\prod_{s\in A}\Gamma \left(\alpha \right)}\;\frac{\prod_{s\in A}\Gamma (\beta n\left({\overset{\leftarrow }{s}}^{R}s\right)+\alpha )}{\Gamma (\beta n\left({\overset{\leftarrow }{s}}^{R}\right)+2\alpha )}\right\}$$

Plugging back into (A1), we have an equation for the posterior probability of the model that produced the data (omitting the normalization factor):

(A7) $$P\left(M_{R}|D_{input,t}\right)=\exp\left[-\gamma |M_{R}|\right]\;\prod_{{\overset{\leftarrow }{s}}^{R}\in A^{R}}\;\left\{\frac{\Gamma (2\alpha )}{\prod_{s\in A}\Gamma \left(\alpha \right)}\;\frac{\prod_{s\in A}\Gamma (n\left({\overset{\leftarrow }{s}}^{R}s\right)+\alpha )}{\Gamma (n\left({\overset{\leftarrow }{s}}^{R}+2\alpha \right))}\right\}$$

Rather than using this equation, however, we considered the log likelihood. After applying a log transformation to Equation (A7), we get a final expression:

(A8) $$\ln\left(P\left(M_{R}|D_{input,t}\right)\right)=-\gamma \left|M_{R}\right|+2^{R}\ln\left[\frac{\Gamma (2\alpha )}{{\left(\Gamma \left(\alpha \right)\right)}^{2}}\right]+\sum_{{\overset{\leftarrow }{s}}^{R}\in A^{R}}\sum_{s\in A}\ln\left[\Gamma \left(n\left({\overset{\leftarrow }{s}}^{R}s\right)+\alpha \right))\right]-\sum_{{\overset{\leftarrow }{s}}^{R}\in A^{R}}\ln[\Gamma (n({\overset{\leftarrow }{s}}^{R})+2\alpha )].$$

Equation (A8) allows the observer to assign relative likelihoods to each order Markov model. Thus, we can analytically obtain a maximum a posteriori order-*R* Markov model. However, for each possible order, the most likely model parameters (transition probabilities) must be estimated before the observer can predict the next symbol. Like before, we use a Dirichlet distribution for the conjugate prior over model probabilities for an order-*R* Markov model:

(A9) $$P\left(\theta |M_{R}\right)=\prod_{{\overset{\leftarrow }{s}}^{R}\in A^{R}}\;\frac{\Gamma (|A|\alpha )}{\prod_{s\in A}\Gamma \left(\alpha \left({\overset{\leftarrow }{s}}^{R}s\right)\right)}\;\delta \left(1-\sum_{s\in A}P(s|{\overset{\leftarrow }{s}}^{R})\right)\;\prod_{s\in A}\;P{\left(s|{\overset{\leftarrow }{s}}^{R}\right)}^{\alpha -1}\;$$

In theory, we could use a different α for every word, so that the Dirichlet prior does not place most of the mass on the uniform distribution. In this paper, we have not done that in order to avoid a proliferation of parameters and overfitting to the data. The utility of the consistency theorem would also be lessened. However, ultimately, the question of what priors to choose is an empirical question, and it is unknown what kind of Dirichlet prior would best describe human subjects. After receiving a time series $D_{input,t}$, substituting A={0,1}⟺|A|=2, and assuming a single concentration hyperparameter α (i.e., the same parameter for all strings, as opposed to a parameter for each string), the observer can obtain the posterior probability distribution over transition probabilities. If the observer drops likelihood updates with probability (1−β), then this posterior distribution is equal to

(A10) $$P\left(\theta |D_{input,t},M_{R}\right)=\prod_{{\overset{\leftarrow }{s}}^{R}\in A^{R}}\;\frac{\Gamma (\beta n\left({\overset{\leftarrow }{s}}^{R}\right)+2\alpha )}{\prod_{s\in A}\Gamma \left(\beta n\left({\overset{\leftarrow }{s}}^{R}s\right)+\alpha \right)}\;\delta \left(1-\sum_{s\in A}P(s|{\overset{\leftarrow }{s}}^{R})\right)\;\prod_{s\in A}\;P{\left(s|{\overset{\leftarrow }{s}}^{R}\right)}^{\beta n({\overset{\leftarrow }{s}}^{R}s)+\alpha -1}.\;$$

For the n-gram argmax strategy, we need to calculate the most likely model parameters. This can calculated as the mode of the posterior distribution lsited in (A10). For each symbol s∈A, this is given by

(A11) $$P_{ML}\left(s|{\overset{\leftarrow }{s}}^{R}\right)=\frac{\alpha +\beta n({\overset{\leftarrow }{s}}^{R}s)-1}{2\alpha +\beta n({\overset{\leftarrow }{s}}^{R})-2}.$$

Since A={0,1}, we only need to calculate this term for one of the two symbols. Using Equations (A8) and (A11), we can can calculate the observer’s expected probability of the next symbol occurring. As described in the text, this is given by

(A12) $$\log\left(L\right)=\sum_{t}\log P_{ML}\left({\hat{s}}_{t+1}|M_{ML,t}\right).$$

This can be calculated using the observer’s string of predictions along with estimated model parameters from the input data.

For the n-gram average strategy, the probability of an observer guessing the symbol *s* in period t+1 is a weighted average of the prediction probabilities over all models and transition probabilities:

(A13) $$P\left({\hat{s}}_{t+1}|D_{input,t}\right)=\sum_{M_{R}\in M}\;\left\{\int d\theta P_{t}\left(\theta ,M_{R}\right)P\left({\hat{s}}_{t+1}|{\overset{\leftarrow }{s}}^{R},\theta ,M_{R}\right)\right\}.$$

From here, note that

(A14) $$P_{t}\left(\theta ,M_{R}|D_{input,t}\right)=\frac{P_{0}\left(M_{R}\right)P_{0}\left(\theta |M_{R}\right)P\left(D_{input,t}|M_{R},\theta \right)}{P(D_{input,t})}.$$

Applying Equation (A14) and the linearity properties of the summation and integration operators to Equation (A13) results in

(A15) $$P\left({\hat{s}}_{t+1}|D_{input,t}\right)=\sum_{M_{R}\in M}P_{0}\left(M_{R}\right)\int d\theta \frac{P_{0}\left(\theta |M_{R}\right)P\left(D_{input,t}|M_{R},\theta \right)}{P(D_{input,t})}$$

where $P_{0}\left(M\right)=e^{-\gamma |M|}$ (where $\left|M\right|=\left(|A\right|-{1)|A|}^{R}=2^{R}$ is the size of the model) is our prior over models. The term in the integral is the probability of observing the input data given the model and transition probabilities, multiplied by the probability of those particular model parameters—integrating this over θ is precisely the expected value of the posterior distribution over model parameters. As such, we can write the expression as

(A16) $$P\left({\hat{s}}_{t+1}|D_{input,t}\right)=\sum_{M_{R}\in M}\left(P_{0}\left(M_{R}\right)\frac{\alpha +\beta n({\hat{s}}_{t+1}|{\overset{\leftarrow }{s}}^{R})}{2\alpha +\beta n({\overset{\leftarrow }{s}}^{R})}\right).$$

With $P({\hat{s}}_{t+1}|D_{input,t})$ in hand, we are well-poised to calculate log likelihoods as described in the main text:

(A17) $$\log\left(L\right)=\sum_{t}\log\langle P\left({\hat{s}}_{t+1}|M_{R,t}\right)\rangle$$
